# Association of urinary sodium and potassium excretion with outcomes of neuroendocrine cancer

**DOI:** 10.3389/fonc.2026.1866503

**Published:** 2026-08-12

**Authors:** Qichao Sun, Mayire Aji, Xu Yan, Yibo Zhang, Fengrui Nan, Yufeng Wang, Lingyu Li

**Affiliations:** 1 Cancer Institute, The First Hospital of Jilin University, Changchun, Jilin, China; 2 Cardiovascular Medicine, The Sixth Affiliated Hospital of Xinjiang Medical University, Urumqi, China; 3 Pathology Department, The First Hospital of Jilin University, Changchun, Jilin, China; 4 Department of Laboratory Medicine, First Hospital of Jilin University, Changchun, Jilin, China; 5 International Center of Future Science, Jilin University, Changchun, Jilin, China; 6 Cancer Center, The First Hospital of Jilin University, Changchun, Jilin, China

**Keywords:** neuroendocrine cancer, STP ratio, UK biobank, urinary potassium, urinary sodium

## Abstract

**Background:**

Sodium and potassium imbalances are pervasive in patients with neuroendocrine cancer (NEC), partly due to syndrome of inappropriate antidiuretic hormone secretion, which impact their prognosis. Whether urinary sodium and potassium excretion is associated with outcomes in patients with NEC remains unknown.

**Methods:**

We conducted a retrospective analysis of 759 patients diagnosed with NEC enrolled in a UK biobank cohort range from 2000 to 2020. Cox proportional hazards models was used to examine the associations between urinary sodium and potassium excretion. Interaction and stratified analyses were conducted including gender, age, NEC primary site, hypertension and sodium intake.

**Results:**

Among the 759 NEC patients, 54.81% were male and 45.19% were female, with a mean age of 52.69 ± 6.11 years. During a median follow-up period of 14.0 months, there were 586 overall deaths and 549 cancer-specific deaths. After adjusting for confounding factors, the urinary sodium-to-potassium ratio (STP) was significantly associated with both overall mortality (HR = 1.16, 95% CI: 1.05-1.28) and cancer-specific mortality (HR = 1.16, 95% CI: 1.05-1.28). When comparing the higher STP group (≥1.29) with the lower STP group (<1.29), the HR was 1.26 (95% CI: 1.04–1.52) for overall mortality and 1.26 (95% CI: 1.04–1.53) for cancer-specific mortality after full adjustment. Additional sensitivity analyses restricted to pulmonary NEC and stratified by primary tumor site showed directionally consistent results.

**Conclusion:**

Higher urinary sodium and lower potassium excretion, as measured by the STP ratio, were associated with increased risks of both overall mortality and cancer-specific mortality in patients with NEC. Our findings suggest that the STP ratio, a parsimonious clinic-ready marker, could provide incremental prognostic information for NEC patients.

## Introduction

Neuroendocrine carcinoma (NEC) is a highly aggressive malignancy arising from neuroendocrine cells, commonly affecting the lungs, gastrointestinal tract, and pancreas (1–3). Beyond its intrinsic tumor biology, NEC frequently disrupts systemic homeostasis, particularly through paraneoplastic syndromes such as the Syndrome of Inappropriate Antidiuretic Hormone Secretion (SIADH) (4, 5). SIADH induces renal water retention and urinary sodium loss, leading to hyponatremia, while simultaneously impairing potassium regulation. These electrolyte disturbances not only exacerbate neurological and cardiovascular complications but may also reflect underlying renal metabolic dysfunction (4, 6), which could influence cancer progression and patient survival.

The kidneys play a central role in maintaining sodium and potassium balance through intricate mechanisms involving the renin-angiotensin-aldosterone system (RAAS), tubular reabsorption, and hormonal regulation (6, 7). In NEC patients, tumor-derived factors (e.g., vasopressin, catecholamines, or ectopic hormones) may disrupt these processes, leading to abnormal urinary sodium and potassium excretion (8). Excessive sodium loss and impaired potassium excretion could indicate renal tubular injury or altered neurohormonal signaling, both of which are associated with worse clinical outcomes in cancer patients (9, 10). Furthermore, chemotherapy agents commonly used in NEC, such as cisplatin, are nephrotoxic and may exacerbate electrolyte imbalances by damaging renal tubules and reducing glomerular filtration efficiency (11, 12).

Urinary electrolyte monitoring offers a non-invasive window into renal metabolic function (13). The sodium-to-potassium ratio (STP) is particularly valuable, as it integrates both excretory processes and is less affected by urine volume variability compared to isolated sodium or potassium measurements (10, 14–18). Prior research has linked elevated STP ratios to hypertension and cardiovascular mortality (10, 19, 20), suggesting that it may also serve as a prognostic marker in cancer patients with renal metabolic disturbances. However, the relationship between urinary sodium/potassium excretion, renal function, and NEC outcomes remains unexplored.

This study leverages data from the UK Biobank to examine whether urinary sodium and potassium excretion patterns, assessed via the STP ratio, correlate with overall and cancer-specific mortality in NEC patients. By incorporating renal metabolic perspectives, we aim to elucidate whether electrolyte imbalances may reflect broader renal dysfunction that contributes to poor prognosis. Our findings could highlight the potential value of renal metabolic monitoring in NEC management and inform interventions—such as dietary sodium restriction, potassium supplementation, or nephroprotective strategies—to mitigate risk and improve survival.

## Materials and methods

### Study population

**Figure 1:**
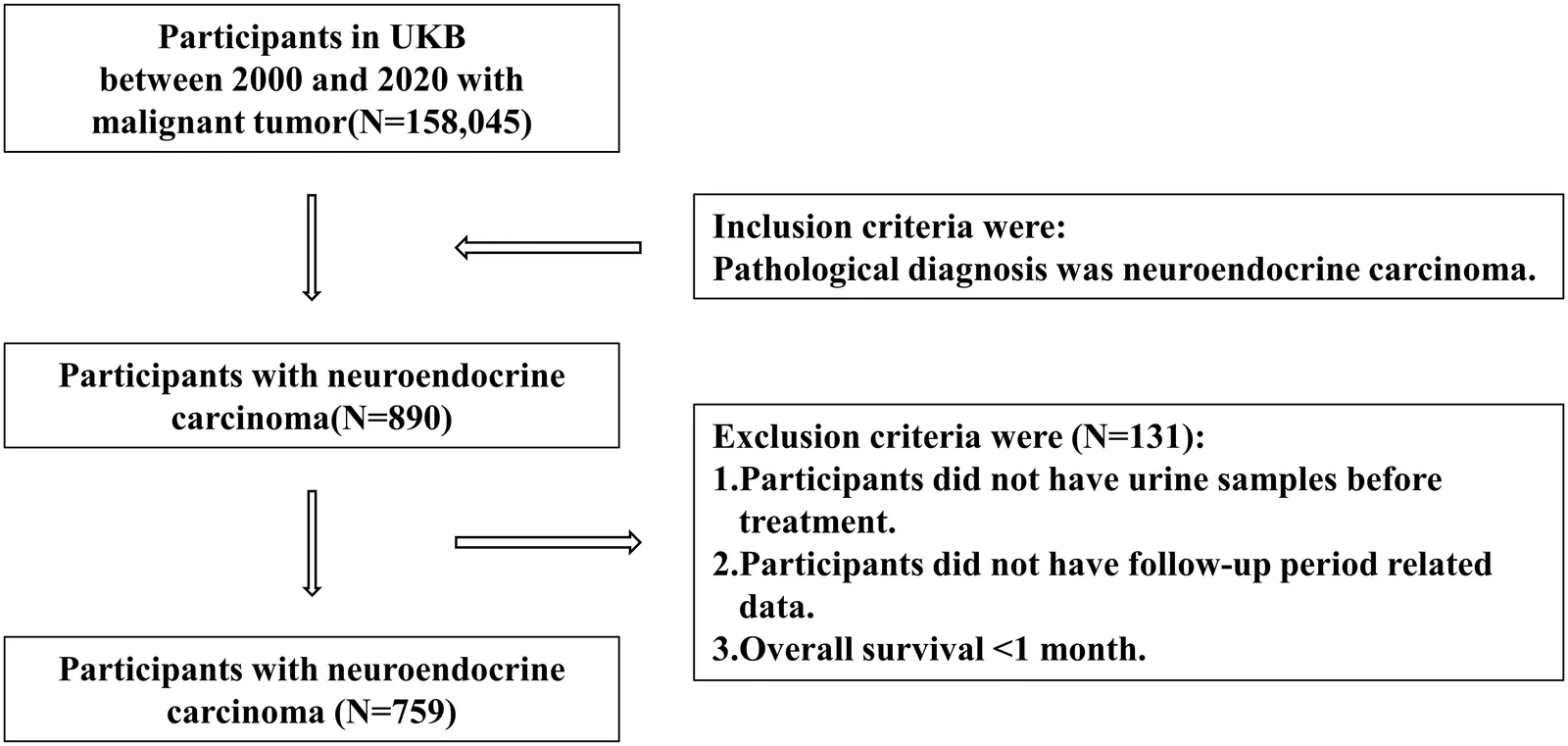
Flowchart of participants. This study analyzed data from 158, 045 malignant tumor patients in the UK Biobank (UKB) from 2000 to 2020. Among these, 890 patients were diagnosed with neuroendocrine cancer. Patients who lacked urine samples before treatment, missing follow-up data, or with an overall survival<1 month were excluded from the analysis. Ultimately, 759 patients were included in the final cohort for this study.

From the UK Biobank (UKB), we identified 158, 045 malignant tumor participants between the January 2000 and October 2020 (**Figure 1**). Among these, 890 participants were diagnosed with NEC. The pathological diagnosis codes are: 8012, 8013, 8041, 8042, 8045, 8240, 8241, 8246 and 8247. Among the included patients, the most common morphology codes were 8041, small cell carcinoma, NOS (N = 455), 8246, neuroendocrine carcinoma (N = 191), and 8012, large cell carcinoma, NOS (N = 56), whereas other morphology codes were less frequent. These distributions indicate that the cohort was mainly composed of small cell carcinoma and neuroendocrine carcinoma, while also including less common neuroendocrine malignancies. In this study, neuroendocrine cancer was operationally defined using UK Biobank morphology codes (ICD10), which captured a spectrum of neuroendocrine malignancies, including small cell carcinoma, large cell neuroendocrine carcinoma, neuroendocrine carcinoma, Merkel cell carcinoma.

Then, we excluded patients due to missing urine samples before treatment, loss of follow-up or the overall survival <1 month. All included participants were followed up until the occurrence of death or the end of follow-up, July 2022, whichever came first. The death event includes overall mortality and deaths where cancer is the underlying cause, even if the immediate cause of death is a complication or comorbidity related to cancer, which is defined as cancer-related mortality events.

### Assessment of urinary sodium and potassium excretion

To investigate the relationship between urinary electrolyte excretion and neuroendocrine carcinoma (NEC) outcomes, we collected urine sodium and potassium data from patients diagnosed with NEC. The UK Biobank (UKB) database codes for urinary sodium and potassium ions are 30530.0.0 and 30520.0.0, respectively. The sodium-to-potassium ratio (STP) was calculated by dividing urinary sodium by urinary potassium at the same time point, measured in mmol/L.

Urinary sodium and potassium were measured from random spot urine samples collected during the UK Biobank assessment visit. The interval between urinary electrolyte measurement and NEC diagnosis was calculated as the urine collection date minus the NEC diagnosis date. Among the 759 participants, urinary sodium and potassium were collected on the same date in 757 participants and within one day in the remaining two participants.

The urinary sodium-to-potassium ratio was analyzed both as a continuous variable and as a median-based binary variable. In the exploratory restricted cubic spline analysis, the value around 1.29 was close to the reference point at which the fitted hazard ratio was set to 1.0. However, because a threshold inferred from visual inspection of a fitted curve may be susceptible to data-driven overfitting, we further adopted a distribution-based approach using the median STP ratio as the cut-off for dichotomized analysis. The median STP ratio in the study population was 1.29411764, which was rounded to 1.29 for presentation. Because this median-based threshold was derived from the empirical distribution of STP rather than from outcome-based optimization or curve fitting, it provides a more transparent and less overfit-prone cut-off for binary classification. Accordingly, STP was primarily analyzed as a continuous variable, while the median-based dichotomized analysis was used as a supportive and exploratory analysis.

### Covariates

This study assessed a range of prognostic factors, including gender, age(age at diagnosis), cardiovascular and cerebrovascular diseases (including angina, stroke, hypertension), diabetes, Body Mass Index, smoking history, histology, surgical history, other malignancies, glucose, glycosylated hemoglobin, type A1c, high-density lipoprotein, insulin-like growth factor 1, triglyceride, cholesterol, urate, creatinine, C-reactive protein, urea and albumin, the utilization of diuretics and sodium intake (Additional salt added to food does not include salt used in cooking or salt contained in the food itself).

The variables can be grouped based on different attributes and characteristics. Here is the categorization of these variables: Basic Demographic Characteristics: Age at diagnosis, Gender; Lifestyle Factors: Smoking history, Sodium intake (additional salt added to food, excluding salt used in cooking or naturally contained in the food); Health Status and Medical History: Body Mass Index (BMI), Diabetes, Cardiovascular and cerebrovascular diseases (including angina, stroke, hypertension), Surgical history, Other malignancies, Utilization of diuretics; Laboratory Indicators: Glucose, HbA1c, High-Density Lipoprotein (HDL), Insulin-like Growth Factor 1 (IGF-1), Triglycerides, Cholesterol, Urate, Creatinine, C-Reactive Protein, Urea, Albumin; Pathological Characteristics: Histology.

### Statistical analysis

This study evaluated the relationship between continuous urinary sodium and potassium excretion, as well as the sodium-to-potassium ratio, with the overall mortality risk and cancer-specific mortality risk in NEC patients using Cox proportional hazards models, adjusting for potential confounders. Survival curves were used to analyze the relationship between the dichotomized STP ratio and overall survival and cancer-specific survival. Values of *p* were two-tailed and significant at values <0.05. All analyses were performed using R 3.3.2 (http://www.R-project.org, The R Foundation) and Free Statistics version 2.3.

## Results

### Study population

We included 759 participants aged 18 years or older who were diagnosed with NEC and registered in the UK Biobank cohort from 2000 to 2020. The median follow-up of 14.0 months. During the follow-up period, there were 586 overall mortality events and 549 cancer-specific mortality events.

Among the 759 participants, the mean age at diagnosis was 66.05 [± 8.14] years (**Table 1**). Of these participants, 416 (54.81%) were male and 343 (45.19%) were female. 62.98% of NEC cases were primary in the lung. The median urinary sodium and potassium excretion tested from random urine samples collected from participants before treatment were 71.1 mmol/L and 56.8 mmol/L, respectively. (The percentile distribution of these values is illustrated in **Supplementary Figure 1**). Specifically, 72 participants (9.49%) had diabetes, with a mean blood glucose level of 5.25 [± 1.38] mmol/L. Cardiovascular and cerebrovascular diseases were present in 343 participants (45.19%). A history of smoking was reported by 643 participants (84.72%), and the mean BMI was 28.41 [± 4.78] kg/m². Among the participants, 549 (72.33%) had a single primary malignant tumor. 98.02% NEC participants (744/759) did not utilize diuretics.

**Table 1:** Baseline characteristics of the study population.

Variable	Whole population
Age at diagnosis (years)	66.05 ± 8.14
Gender n (%)	
Male	416(54.81)
Female	343(45.19)
Urinary sodium (mmol/L)	71.1
Urinary potassium (mmol/L)	56.8
Diabetes n (%)	
Yes	72(9.49)
No	683(89.99)
Unknown	4(0.52)
Blood glucose (mmol/L)	5.25 ± 1.38
Cardiovascular and Cerebrovascular diseases n (%)	
Yes	343(45.19)
No	412(54.28)
Unknown	4(0.53)
Smoke n (%)	
Yes	643(84.72)
No	115(15.15)
Unknown	1(0.13)
BMI (kg/m²)	28.41 ± 4.78
HDL (mmol/L)	1.34 ± 0.37
IGF.1(nmol/L)	20.30 ± 6.56
Triglycerides (mmol/L)	1.95 ± 1.04
Cholesterol (mmol/L)	5.43 ± 1.23
Creatinine (mmol/L)	74.86 ± 29.73
Albumin (g/L)	44.57 ± 2.63
Combined with other malignant tumors n (%)	
0	549(72.33)
1	174(22.92)
2	35(4.61)
Unknown	1(0.13)
NEC primary site n (%)	
Lung	480(62.98)
Small intestine	32(4.21)
Pancreas	31(4.08)
Bladder	27(3.56)
Colon	25(3.29)
Skin	20(2.63)
Esophagus	19(2.50)
Liver	17(2.24)
Rectum	13(1.71)
Others	90(11.86)
Sodium intake n (%)	
Never	342(45.06)
Sometimes	220(28.99)
Usually/Always	197(25.95)
Diuretics n (%)	
Not used	744(98.02)
Hydrochlorothiazide	4(0.52)
Furosemide	11(1.45)

Plus–minus values are means ± SD. Percentages may not total 100 because of rounding.

BMI, Body Mass Index (the weight in kilograms divided by the square of the height in meters); HDL, high density lipoprotein; IGF.1, Insulin-Like Growth Factor 1; Cardiovascular and Cerebrovascular diseases: including angina, stroke, hypertension; Sodium intake: Additional salt added to food does not include salt used in cooking or salt contained in the food itself.

### Association of urinary sodium and potassium excretion with overall mortality and cancer-specific mortality

To investigate the risk factors associated with mortality in patients with NEC, we employed a univariate Cox regression model to examine the impact of urinary sodium and potassium excretion. The analysis revealed that urinary sodium excretion was significantly associated with an increased overall mortality risk (HR = 1.03, 95% CI: 1.01-1.05, *p* = 0.003) and cancer-specific mortality risk (HR = 1.01, 95% CI: 1.00-1.03, *p* = 0.184). (**Supplementary Table 1**) An increase of 10 mmol/L in urinary sodium excretion is associated with a 3% rise in overall mortality risk and a 1% rise in cancer-specific mortality risk. Conversely, urinary potassium excretion did not show a statistically significant association with either overall mortality risk (HR = 0.99, 95% CI: 0.97-1.01, *p* = 0.197) or cancer-specific mortality risk (HR = 0.98, 95% CI: 0.96-1.01, *p* = 0.19). We then conducted further analysis using multivariate Cox regression models to adjust for potential confounding variables identified in the univariate analysis. Post-adjustment, urinary sodium excretion remained significantly associated with overall mortality risk (HR = 1.02, 95% CI: 1.00-1.05, *p* = 0.049) but not with cancer-specific mortality risk (HR = 1.01, 95% CI: 0.99-1.03, *p* = 0.406). Urinary potassium excretion continued to show no significant association with overall mortality risk (HR = 0.99, 95% CI: 0.96-1.02, *p* = 0.384) or cancer-specific mortality risk (HR = 0.97, 95% CI: 0.95-1.01, *p* = 0.105).

### High STP ratio was associated with increased risks of both overall mortality and cancer-specific mortality

**Figure 2:**
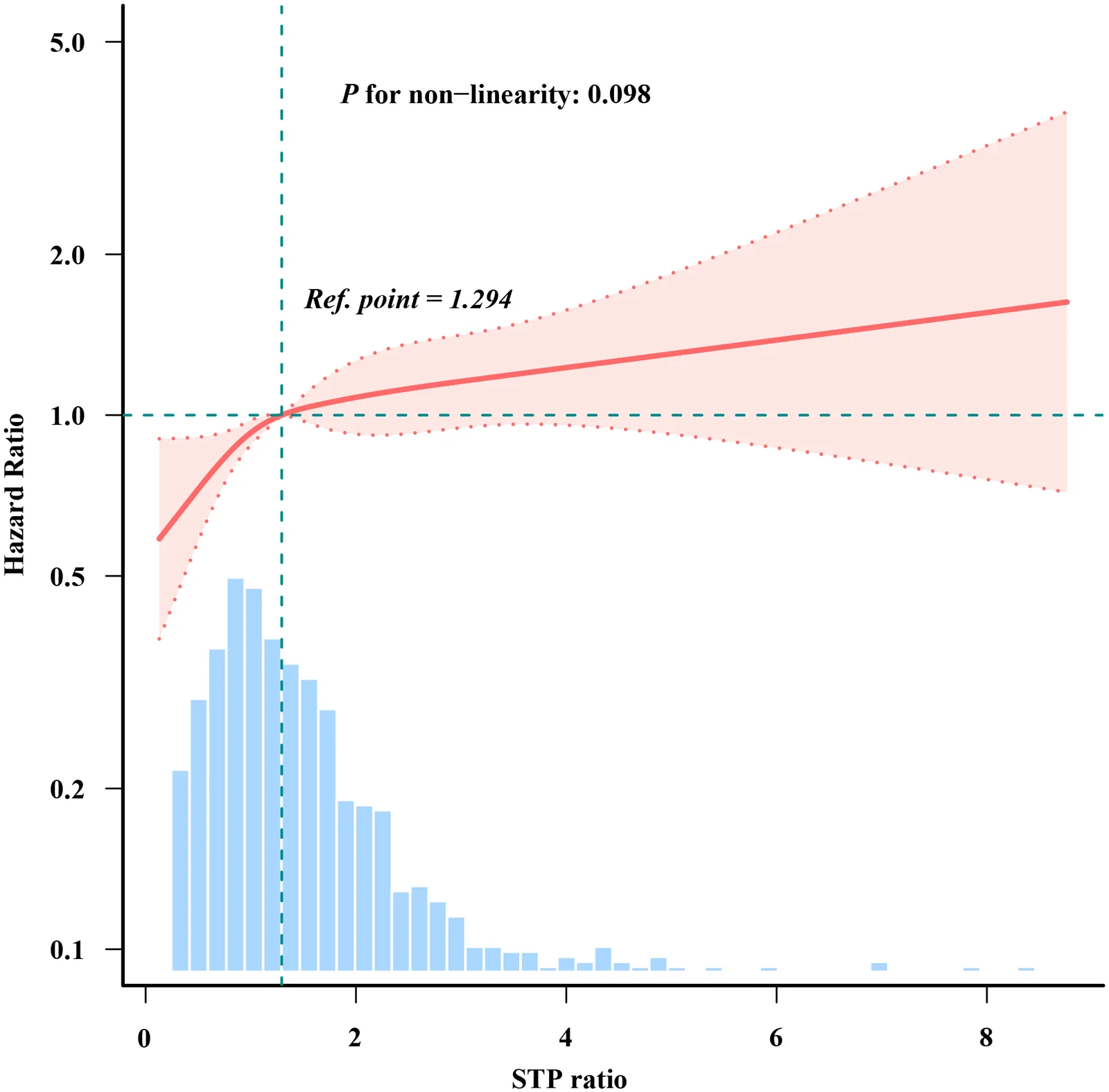
Fitted curve of the risk relationship between urinary STP ratio and overall mortality. This plot depicts the relationship between the urinary sodium-to-potassium ratio (STP) and the hazard ratio (HR) for overall mortality. The horizontal axis represents the STP ratio, while the vertical axis represents the HR. The red line shows the fitted HR, with the shaded area indicating the 95% confidence interval. The median STP ratio of the study population was 1.294 and was used as the reference value in the restricted cubic spline model, where the HR was set to 1.0. This value was rounded to 1.29 for the median-based dichotomized analysis. The blue bars at the bottom represent the distribution of STP ratios among the study participants.

**Figure 3:**
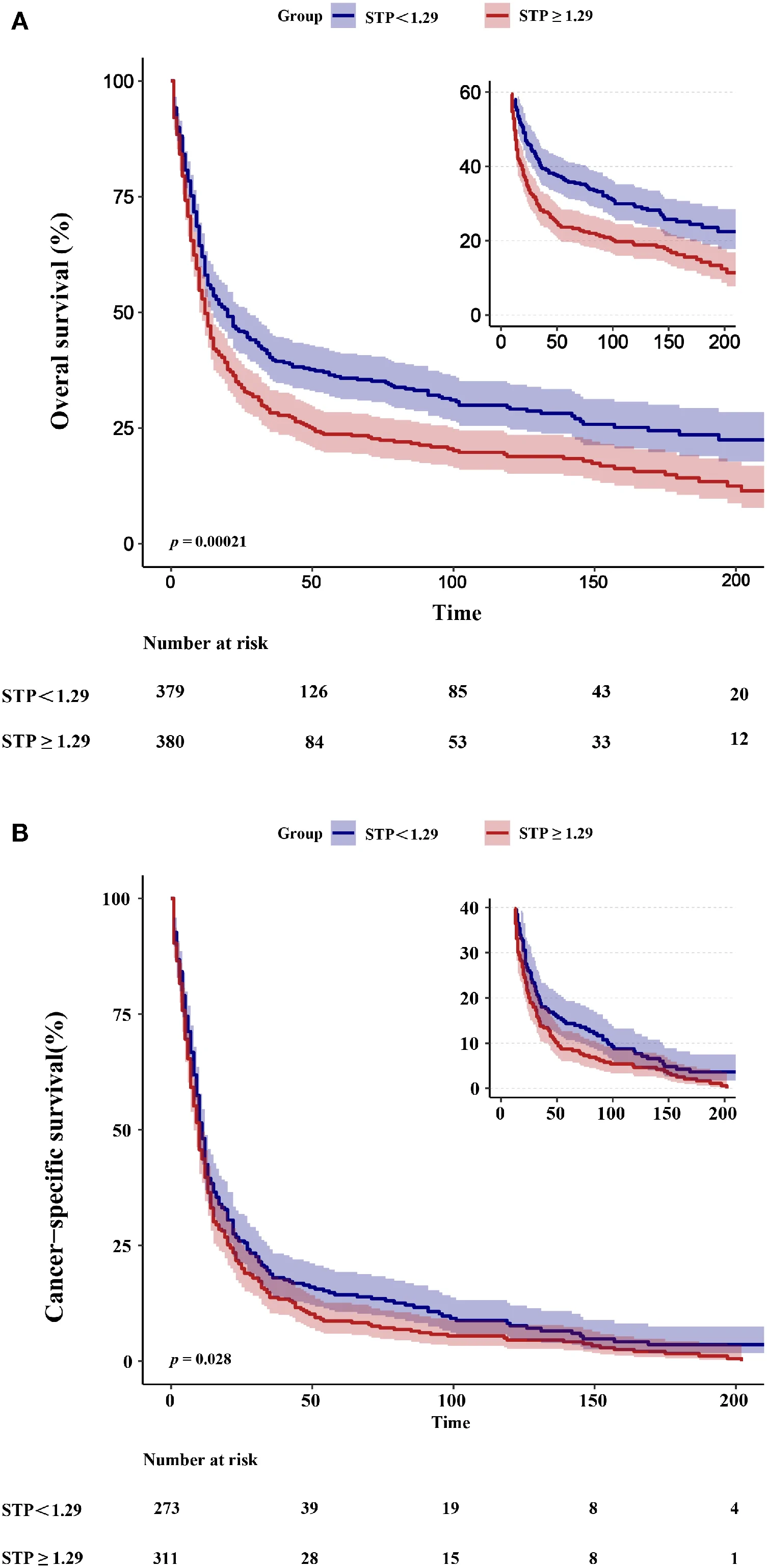
Kaplan-Meier curves for survival based on STP ratio in patients with NEC. Association between STP ratio and overall survival **(A)** and cancer-specific survival **(B)** in patients with NEC. Kaplan-Meier survival curves comparing overall survival or cancer-specific survival between participants with an STP ratio <1.29 (blue line) and those with an STP ratio ≥1.29 (red line). The shaded areas represent the 95% confidence intervals. The number of participants at risk at various time points is displayed below the main plot.

To achieve a more comprehensive risk assessment and enhance clinical relevance, this study analyzed the association between the sodium-to-potassium ratio (STP) and mortality risk. Our analysis suggested a generally increasing trend in mortality risk with higher STP ratio, without strong evidence of non-linearity (*P* for non-linearity = 0.098; **Figure 2**). The reference value in the spline model was the median STP ratio of 1.29411764, which was also rounded to 1.29 for the median-based dichotomized analysis. Using Kaplan-Meier curves to compare the survival of the two groups over the same follow-up period, it is evident that both the overall survival rate and cancer-specific survival rate are lower in the group with an STP ratio ≥1.29 (**Figure 3**). Due to the high proportion of deaths, the difference in median overall survival between the two groups is not significant, so the 75% death occurrence time was adopted. The median survival time for overall survival is 22 months, and the median survival time for cancer-specific survival is 20 months. (with an average overall survival of 23.94 months and an average cancer-specific survival of 20.95 months).

Subsequently, we used the 1.29 as the cut-off to categorize STP groups (**Table 2**). Among the 586 total recorded deaths, each unit increase in the STP ratio was associated with a 17% increase in mortality risk in the crude model (HR = 1.13, 95% CI: 1.06-1.22). This significant association persisted in the adjusted model (HR = 1.17, 95% CI: 1.06-1.28). In the median-based dichotomized analysis, participants with STP ≥1.29 had a significantly higher risk of overall mortality than those with STP <1.29 after adjustment for all covariates (HR = 1.26, 95% CI: 1.04–1.52).

**Table 2:** Association of sodium-to-potassium excretion with overall mortality and cancer-specific mortality.

Variable	Total no.	Events (n%)	Model 1 HR(95%CI)	Model 2 HR(95%CI)	Model 3 HR(95%CI)	Model 4 HR(95%CI)	Model 5 HR(95%CI)	Model 6 HR(95%CI)
Overall mortality								
STP ratio	759	586(77.2)	1.13 (1.06~1.22)	1.15 (1.07~1.24)	1.17 (1.09~1.26)	1.15 (1.07~1.23)	1.17 (1.06~1.28)	1.17 (1.06~1.28)
Median-based urinary STP ratio category								
<1.29	377	272(46.4)	Ref	Ref	Ref	Ref	Ref	Ref
≥1.29	382	314(53.6)	1.36 (1.15~1.6)	1.39 (1.18~1.63)	1.38 (1.17~1.63)	1.33 (1.12~1.57)	1.25 (1.04~1.51)	1.26 (1.04~1.52)
Cancer-specific mortality								
STP ratio	759	549(72.3)	1.15 (1.07~1.24)	1.17 (1.09~1.26)	1.19 (1.10~1.28)	1.16 (1.08~1.25)	1.17 (1.07~1.29)	1.17 (1.07~1.29)
Median-based urinary STP ratio category								
<1.29	377	250 (45.5)	Ref	Ref	Ref	Ref	Ref	Ref
≥1.29	382	299 (54.5)	1.39 (1.18~1.65)	1.41 (1.19~1.67)	1.42 (1.19~1.68)	1.36 (1.14~1.61)	1.25 (1.03~1.52)	1.26 (1.04~1.53)

This table presents the hazard ratios (HR) and 95% confidence intervals (CI) for the association between the sodium-to-potassium (STP) ratio and both overall mortality and cancer-specific mortality among 759 participants. The data is adjusted across six different models. The HR for overall mortality and cancer-specific mortality is provided for the continuous variable of STP ratio and the binary classification of STP ratio, with <1.29 as the reference group. The HRs range from 1.13 to 1.17 across models for overall mortality. The HRs range from 1.15 to 1.19 across models for cancer-specific mortality. Compared with the reference group (<1.29), showing increased HRs for higher STP ratios. The table highlights significant associations and trends between higher STP ratios and increased risks of both overall and cancer-specific mortality.

Model 1 was crude model; Model 2 was adjusted by age at diagnosis, gender; Model 3: Model 2+BMI, diabetes, smoking history, (including angina, stroke, hypertension);Model 4: Model 3+histology, surgical history, merging other malignant tumors; Model 5: Model 4+ glucose +HbA1c.+ HDL +IGF.1+ triglyceride + cholesterol + urate + creatinine + C-reactive protein + urea + albumin; Model 6: Model 5+ the utilization of diuretics + sodium intake (Additional salt added to food does not include salt used in cooking or salt contained in the food itself).

To assess the cancer-specific deaths. In the crude model, each unit increase in the STP ratio was associated with a 7% increase in cancer-specific mortality risk (HR = 1.15, 95% CI: 1.07-1.24). This association remained significant, with each unit increase in the STP ratio linked to a 17% increase in cancer-specific mortality risk (HR = 1.17, 95% CI: 1.07-1.29), when adjusted by all covariates.Similarly, in the median-based dichotomized analysis, participants with STP ≥1.29 had a significantly higher risk of cancer-specific mortality than those with STP <1.29 after adjustment for all covariates (HR = 1.26, 95% CI: 1.04–1.53).

Given that 343 (45.2%) of the 759 participants had pre-existing cardiovascular disease, we conducted a Fine-Gray competing risk analysis to account for cardiovascular death as a competing event. (**Supplementary Table 2**) This analysis affirmed that a higher urinary STP ratio was independently associated with an increased risk of cancer-specific mortality. In the fully adjusted model (Model 6), each one-unit increment in the STP ratio was associated with a 16% increase in the sub distribution hazard (sHR = 1.16, 95% CI: 1.06–1.26). Similarly, the high-STP group (≥1.29) exhibited a 22% higher cumulative incidence of cancer-specific death compared to the low-STP group (sHR = 1.22, 95% CI: 1.01–1.47).

These findings suggest that higher urinary STP ratio remained associated with cancer-specific mortality after accounting for competing cardiovascular death. However, given the observational design and potential residual confounding, this result should be interpreted as an association rather than evidence of causality.

### Multivariable stratified analysis for STP ratio

**Figure 4:**
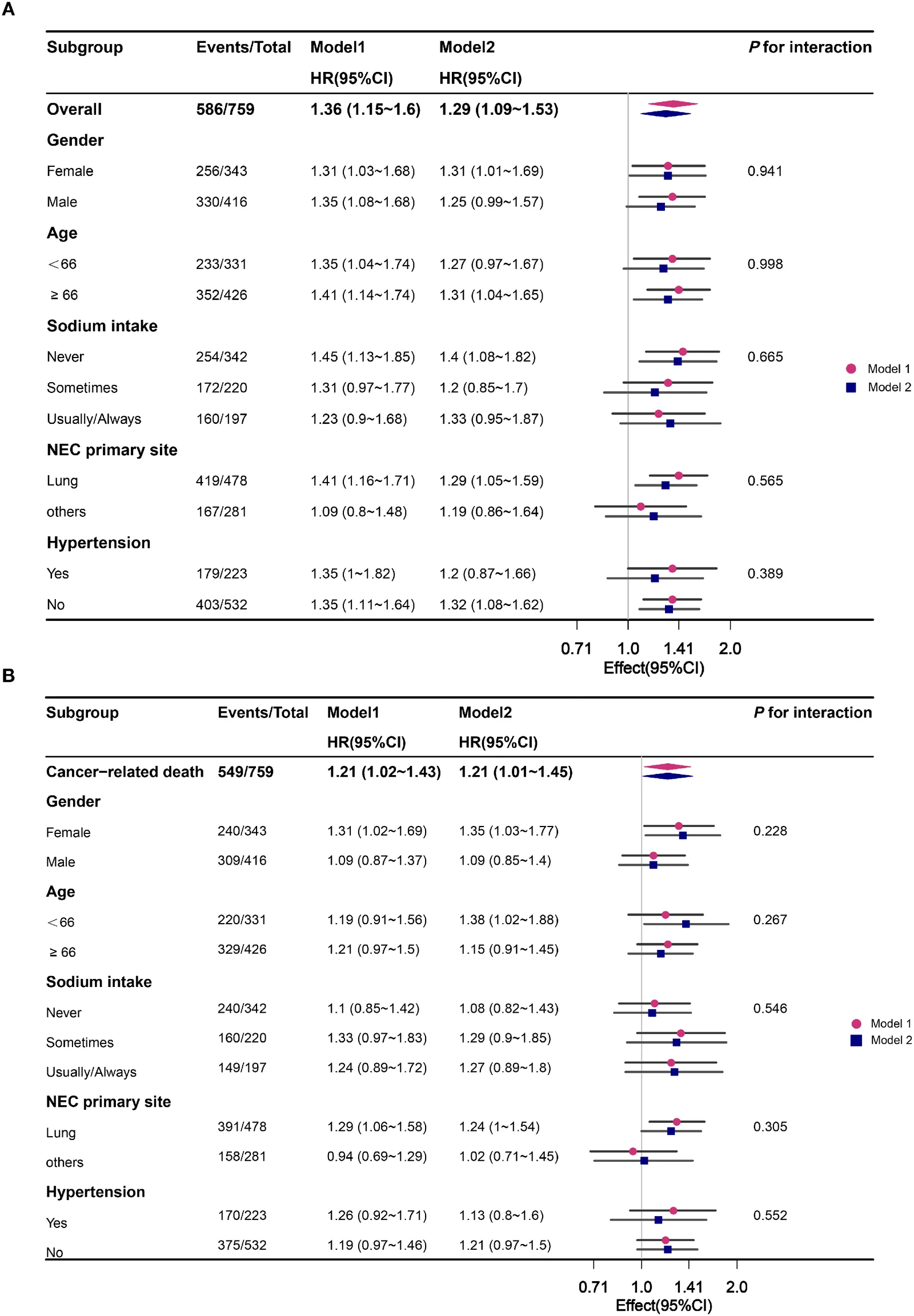
Forest plots for the associations of urinary STP ratio with overall mortality and cancer-specific mortality. This forest plot illustrates the hazard ratios (HR) for overall mortality **(A)** and cancer-specific mortality **(B)** across various subgroups in both Model 1 and Model 2. The subgroups are defined by gender, age, sodium intake (additional salt added to food does not include salt used in cooking or salt contained in the food itself), NEC primary site and hypertension. Lines represent the 95% confidence intervals **(CI)** of the hazard ratio (HR) for each subgroup. The *P* for interaction values indicate the significance of the differences between subgroups. Model 1: crude model; Model 2: adjusted by BMI, smoking history, histology, surgical history, combined with other malignant tumors and the utilization of diuretics.

To determine whether the STP ratio exhibits subgroup-specific or population-specific distributions concerning overall mortality risk and cancer-related mortality risk, we conducted subgroup analyses based on gender, age at diagnosis, additional salt intake, NEC primary site, and hypertension. The forest plot in **Figure 4** shows the group with STP < 1.29 as the reference, with data for STP ≥ 1.29 presented. The analyses revealed no significant interactions between subgroups, with the high STP group (≥ 1.29) consistently showing higher risk in overall death (**Figure 4A**) or cancer-specific death (**Figure 4B**), compared to the low STP (< 1.29) group. Additionally, compared to the low STP group, the high STP group showed a significantly higher risk of overall mortality and cancer-related mortality in the pulmonary primary NEC subgroup, whereas no significant difference was observed in patients with NEC from other organs.

In subsequent analyses, additional adjustments were made for BMI, smoking history, histology, surgical history, merging other malignant tumors and the utilization of diuretics. This study demonstrates that, after adjusting for different categorical variables, the fundamental linear relationship between a higher STP ratio and mortality risk remained robust.

### Sensitivity analyses according to primary tumor site

Because pulmonary NEC accounted for the majority of cases in the study cohort, additional analyses were performed to evaluate whether the association between urinary STP ratio and mortality outcomes differed according to primary tumor location. Given the potential physiological relevance of the lung to electrolyte regulation through the renin–angiotensin–aldosterone system (RAAS), pulmonary and non-pulmonary NEC were further examined separately.

First, a sensitivity analysis restricted to patients with pulmonary NEC was conducted (**Supplementary Table 3**). In this subgroup, continuous STP remained significantly associated with both overall mortality and cancer-specific mortality in the fully adjusted Model 6. Each one-unit increase in STP was associated with an increased risk of overall mortality (HR = 1.17, 95% CI: 1.05–1.31, P = 0.006) and cancer-specific mortality (HR = 1.17, 95% CI: 1.05–1.32, P = 0.006).

Second, stratified analyses were performed to compare pulmonary and non-pulmonary NEC (**Supplementary Table 4**). The association between STP and mortality outcomes appeared more evident among patients with pulmonary NEC. In patients with non-pulmonary NEC, the estimated hazard ratios showed a similar direction of association but did not reach statistical significance, likely reflecting the smaller sample size and reduced statistical power in this subgroup. Formal interaction analyses in the fully adjusted model did not demonstrate significant effect modification by primary tumor site (*P* for interaction = 0.567 for overall mortality and *P* = 0.648 for cancer-specific mortality).

Overall, these findings indicate that the association between urinary STP ratio and mortality outcomes remained observable in pulmonary NEC and appeared stronger in this subgroup; however, no statistically significant difference was identified between pulmonary and non-pulmonary NEC.

## Discussion

This study provides novel evidence that urinary sodium and potassium excretion patterns, as reflected by the STP ratio, are significantly associated with survival outcomes in patients with NEC. The robust association between higher STP ratios and increased mortality risk persisted across multiple adjusted models, suggesting these electrolyte imbalances may represent more than just epiphenomena of advanced disease. Importantly, the STP ratio emerged as a stronger predictor than individual electrolyte measurements, highlighting the clinical value of assessing these minerals in combination rather than isolation. These findings extend previous research on electrolyte disturbances in cancer patients by specifically demonstrating their prognostic relevance in NEC, a population particularly prone to SIADH and related metabolic complications.

From a renal pathophysiology perspective, the observed associations likely reflect multiple interconnected mechanisms. The kidneys’ impaired ability to appropriately regulate sodium and potassium in NEC patients may stem from both tumor-related factors and treatment effects (11, 21, 22). SIADH, frequently seen in NEC, promotes inappropriate water retention and sodium loss through its effects on renal collecting ducts (23). Concurrently, many NEC patients receive platinum-based chemotherapies known to cause tubular injury, potentially disrupting potassium secretion and further exacerbating electrolyte imbalances (24, 25). The stronger association seen in pulmonary NEC aligns with the higher prevalence of SIADH in lung neuroendocrine tumors (26), suggesting primary tumor location may influence the severity of renal metabolic dysfunction (27). These disturbances may create a vicious cycle where electrolyte imbalances worsen patient performance status while simultaneously creating a more permissive environment for tumor progression (28, 29).

The clinical implications of these findings are potentially significant. While 24-hour urine collections remain the gold standard for electrolyte assessment (15), our results support the use of spot urine STP measurements as a practical alternative in busy oncology practices (30, 31). However, the present study did not directly evaluate any intervention, and the observational design does not support conclusions that modifying sodium or potassium balance would improve survival in patients with NEC. Therefore, urinary STP should be interpreted as a potential observational marker that may help identify patients with altered renal-electrolyte homeostasis and higher mortality risk, rather than as a tool to directly guide therapeutic intervention. Prospective and interventional studies are needed before any STP-guided clinical strategy can be recommended.

The biological heterogeneity of neuroendocrine malignancies also warrants consideration. Using UK Biobank morphology codes, the present cohort captured a broad spectrum of entities, including small cell carcinoma, large-cell neuroendocrine carcinoma, neuroendocrine carcinoma, Merkel cell carcinoma (32). Because pulmonary neuroendocrine carcinoma accounted for most cases, the observed association may primarily reflect risk patterns in pulmonary neuroendocrine malignancies rather than across all neuroendocrine tumor types. Lung neuroendocrine tumors themselves comprise heterogeneous entities, including high-grade small cell carcinoma and large-cell neuroendocrine carcinoma. One possible explanation is the distinctive pulmonary RAAS milieu, as the pulmonary vascular endothelium is a major site of angiotensin-converting enzyme activity and may contribute to RAAS-mediated electrolyte regulation (33, 34). However, ACE activity, angiotensin II, renin, aldosterone, and tumor-specific RAAS activity were not measured in this study. This proposed mechanism therefore remains speculative and should be tested in future studies incorporating tumor-site-specific and pathway-level measurements.

Several limitations should be acknowledged when interpreting these results. The single measurement of urinary electrolytes may not fully capture a patient’s typical sodium and potassium excretion patterns (35), potentially leading to misclassification. Although sodium intake was adjusted for in the fully adjusted model, the UK Biobank sodium intake variable reflects additional salt added to food and does not fully capture total dietary sodium intake; therefore, residual confounding related to dietary sodium intake cannot be completely excluded. In addition, several important oncological variables were unavailable, including tumor stage, grade/Ki-67 index, metastatic burden, performance status, systemic treatment, platinum exposure, and baseline sodium abnormalities such as hyponatremia or SIADH. These variables may influence both urinary STP ratio and survival outcomes; therefore, residual confounding and reverse causality cannot be fully excluded. The observational nature of the study precludes definitive conclusions about causality, and it remains unclear whether modifying STP ratios would actually improve survival. Additionally, the generalizability of findings may be limited by the use of UK Biobank data, which comes from a predominantly white population with potentially different dietary patterns than other ethnic groups.

Future research directions should focus on validating these findings in independent NEC cohorts, particularly those with more diverse patient populations. Mechanistic studies exploring how sodium and potassium imbalances might directly influence tumor biology could provide important insights, as could investigations into whether specific treatments affect electrolyte excretion patterns. Prospective studies examining the impact of STP-guided interventions on patient outcomes would be particularly valuable. Additionally, research exploring the potential interplay between STP ratios and other prognostic factors in NEC, such as tumor grade or proliferation index, could help refine risk stratification approaches.

In conclusion, this study identifies urinary sodium-to-potassium ratio as a promising prognostic marker in neuroendocrine carcinoma that reflects underlying renal metabolic dysfunction. Higher urinary STP ratio was associated with poorer overall survival and increased cancer-specific mortality, both when STP was analyzed as a continuous variable and when it was categorized using the median-based cut-off. While further research is needed to validate these findings, establish causality, and evaluate potential interventions, our results highlight the importance of renal-electrolyte homeostasis in NEC patients.

## Data Availability

The datasets presented in this study can be found in online repositories. The names of the repository/repositories and accession number(s) can be found below: http://www.ukbiobank.ac.uk/Researchers can apply for access to the data for health-related research that is in the public interest. All analyses in the present study were conducted under UK Biobank Application ID [84347].

## Supplementary material

Supplementary Table 1 Association of urinary sodium and potassium excretion with overall mortality and cancer-specific mortality.

Supplementary Table 2 Association of sodium-to-potassium excretion with cancer-specific mortality in competing-risk analysis.

Supplementary Table 3 Restricted analysis in pulmonary NEC.

Supplementary Table 4 Stratified analysis by primary tumor site: pulmonary**(A)** versus non-pulmonary NEC**(B)**.

Supplementary Figure 1 Percentile distribution of urinary sodium and potassium excretion.
