# Supplementary material for: Association of urinary sodium and potassium excretion with outcomes of neuroendocrine cancer
Source: Front Oncol. 2026 Aug 12;16:1866503. doi: 10.3389/fonc.2026.1866503 (PMC13508161; doi:10.3389/fonc.2026.1866503)
Supplement: Supplementary file 1 [file DataSheet1.pdf]

Supplementary Table 1. Association of Urinary Sodium and Potassium Excretion with Overall Mortality and Cancer-specific Mortality.

Variable	Total No.	Events (n%)	Model 1 HR(95%CI)	Model 2 HR(95%CI)	Model 3 HR(95%CI)	Model 4 HR(95%CI)	Model 5 HR(95%CI)	Model 6 HR(95%CI)
**Overall mortality**								
Sodium (per 10mmol/L increase)	759	586(77.2)	1.03 (1.01~1.05)	1.03 (1.01~1.05)	1.03 (1.01~1.05)	1.02 (1~1.03)	1.02 (1~1.04)	1.02 (1~1.05)
Potassium	759	586(77.2)	0.99 (0.97~1.01)	0.98 (0.96~1.01)	0.98 (0.96~1.01)	0.98 (0.95~1)	0.99 (0.96~1.02)	0.99 (0.96~1.02)
**Cancer-specific mortality**								
Sodium (per 10mmol/L increase)	759	549(72.3)	1.01 (1~1.03)	1.01 (0.99~1.03)	1.01 (0.99~1.03)	1.01 (0.99~1.03)	1.01 (0.99~1.03)	1.01 (0.99~1.03)
Potassium	759	549(72.3)	0.98 (0.96~1.01)	0.98 (0.96~1.01)	0.98 (0.96~1.01)	0.98 (0.95~1)	0.98 (0.95~1.01)	0.97 (0.95~1.01)

This table presents the hazard ratios (HR) and 95% confidence intervals (CI) for the association between urinary sodium and potassium excretion and both overall mortality and cancer-specific mortality among 759 participants, adjusted across six different models. For overall mortality, each 10 mmol/L increase in sodium excretion corresponds to HRs ranging from 1.02 to 1.03, indicating a small but consistent increase in overall mortality risk. In contrast, potassium excretion has HRs ranging from 0.98 to 0.99, showing no significant association with overall mortality risk. For cancer-specific mortality, each 10 mmol/L increase in sodium excretion corresponds to HRs ranging from 1.01 to 1.02, suggesting a modest increase in cancer-specific mortality risk. Potassium excretion has HRs ranging from 0.97 to 0.98, indicating no significant association with cancer-specific mortality risk.

Model 1 was crude model; Model 2 was adjusted by age at diagnosis, gender; Model 3: Model 2+BMI, diabetes, smoking history, cardiovascular and cerebrovascular diseases (including angina, stroke, hypertension);Model 4: Model 3+histology, surgical history, merging other malignant tumors; Model 5: Model 4+ glucose +HbA1c.+ HDL +IGF.1+ triglyceride + cholesterol + urate + creatinine + C-reactive protein + urea + albumin; Model 6: Model 5+ the utilization of diuretics + sodium intake (Additional salt added to food does not include salt used in cooking or salt contained in the food itself).

Supplementary Table 2. Association of Sodium-to-Potassium Excretion with cancer-specific mortality in competing-risk analysis.

Variable	Total No.	Events (n%)	Model 1 HR(95%CI)	Model 2 HR(95%CI)	Model 3 HR(95%CI)	Model 4 HR(95%CI)	Model 5 HR(95%CI)	Model 6 HR(95%CI)
**Cancer-specific mortality**								
STP ratio	736	549(74.6)	1.14 (1.06~1.22)	1.16 (1.08~1.24)	1.18 (1.1~1.26)	1.13 (1.04~1.22)	1.15 (1.05~1.26)	1.16 (1.06~1.26)
Median-based urinary STP ratio category								
<1.29	363	250 (68.9)	Ref	Ref	Ref	Ref	Ref	Ref
≥1.29	373	299 (80.2)	1.36 (1.15~1.6)	1.36 (1.15~1.61)	1.34 (1.13~1.6)	1.24 (1.04~1.48)	1.2 (1~1.45)	1.22 (1.01~1.47)

This table presents the Fine–Gray sub distribution hazards ratios (HR) and 95% confidence intervals (CI) for the association between the sodium-to-potassium (STP) ratio and cancer-specific mortality after accounting for cardiovascular death as a competing event, among 759 participants. The data is adjusted across six different models. The HR for cancer-specific mortality is provided for the continuous variable of STP ratio and the binary classification of STP ratio, with <1.29 as the reference group. The HRs range from 1.2 to 1.36 across models for cancer-specific mortality. Compared with the reference group (<1.29), showing increased HRs for higher STP ratios. The table highlights significant associations and trends between higher STP ratios and increased cancer-specific mortality, with cardiovascular death as a competing event.

Model 1 was crude model; Model 2 was adjusted by age at diagnosis, gender; Model 3: Model 2+BMI, diabetes, smoking history, cardiovascular and cerebrovascular diseases (including angina, stroke, hypertension);Model 4: Model 3+histology, surgical history, merging other malignant tumors; Model 5: Model 4+ glucose +HbA1c.+ HDL +IGF.1+ triglyceride + cholesterol + urate + creatinine + C-reactive protein + urea + albumin; Model 6: Model 5+ the utilization of diuretics + sodium intake (Additional salt added to food does not include salt used in cooking or salt contained in the food itself).

Supplementary Table 3. Restricted analysis in pulmonary NEC.

Variable	Total No.	Events (n%)	Model 1 HR(95%CI)	Model 2 HR(95%CI)	Model 3 HR(95%CI)	Model 4 HR(95%CI)	Model 5 HR(95%CI)	Model 6 HR(95%CI)
**Overall mortality**								
STP ratio	480	419(87.3)	1.12 (1.04-1.21)	1.10 (1.01-1.19)	1.13 (1.04-1.23)	1.12 (1.03-1.22)	1.17 (1.05-1.32)	1.17 (1.05-1.31)
Median-based urinary STP ratio category								
<1.29	217	180(43.0)	Ref	Ref	Ref	Ref	Ref	Ref
≥1.29	263	239(57.0)	1.41 (1.16-1.71)	1.33 (1.09-1.61)	1.35 (1.11-1.65)	1.29 (1.05-1.58)	1.17 (0.92-1.48)	1.18 (0.93-1.50)
**Cancer-specific mortality**								
STP ratio	480	391(81.5)	1.13 (1.04-1.22)	1.11 (1.02-1.21)	1.14 (1.05-1.24)	1.13 (1.04-1.24)	1.18 (1.05-1.32)	1.17 (1.05-1.32)
Median-based urinary STP ratio category								
<1.29	217	165 (42.2)	Ref	Ref	Ref	Ref	Ref	Ref
≥1.29	263	226 (57.8)	1.42 (1.16-1.74)	1.35 (1.10-1.65)	1.37 (1.12-1.69)	1.30 (1.05-1.60)	1.16 (0.91-1.47)	1.17 (0.91-1.49)

This table presents the hazard ratios (HR) and 95% confidence intervals (CI) for the association between the urinary sodium-to-potassium (STP) ratio and both overall mortality and cancer-specific mortality among 480 patients with pulmonary neuroendocrine cancer (NEC). The data were adjusted across six different models. The HRs for overall mortality and cancer-specific mortality are provided for both the continuous STP ratio and the median-based binary classification of STP ratio, with STP <1.29 as the reference group. Among pulmonary NEC patients, 217 participants had an STP ratio <1.29 and 263 participants had an STP ratio ≥1.29. During follow-up, 419 overall mortality events and 391 cancer-specific mortality events were observed. For the continuous STP ratio, the HRs ranged from 1.10 to 1.17 across models for overall mortality and from 1.11 to 1.18 across models for cancer-specific mortality. In the fully adjusted Model 6, continuous STP remained significantly associated with both overall mortality (HR = 1.17, 95% CI: 1.05–1.31) and cancer-specific mortality (HR = 1.17, 95% CI: 1.05–1.32). For the dichotomized STP ratio, higher STP was associated with elevated HRs in the crude and partially adjusted models, whereas the associations were attenuated and did not reach statistical significance in the fully adjusted Model 6. These findings suggest that the association between STP ratio and mortality outcomes remained evident among patients with pulmonary NEC, particularly when STP was modeled as a continuous variable.

Model 1 was crude model; Model 2 was adjusted by age at diagnosis, gender; Model 3: Model 2+BMI, diabetes, smoking history, cardiovascular and cerebrovascular diseases (including angina, stroke, hypertension);Model 4: Model 3+histology, surgical history, merging other malignant tumors; Model 5: Model 4+ glucose +HbA1c.+ HDL +IGF.1+ triglyceride + cholesterol + urate + creatinine + C-reactive protein + urea + albumin; Model 6: Model 5+ the utilization of diuretics + sodium intake (Additional salt added to food does not include salt used in cooking or salt contained in the food itself).

Supplementary Table 4. Stratified analysis by primary tumor site: pulmonary(A) versus non-pulmonary NEC(B).

A. In the pulmonary NEC subgroup.

Variable	Total No.	Events (n%)	Model 1 HR(95%CI)	Model 2 HR(95%CI)	Model 3 HR(95%CI)	Model 4 HR(95%CI)	Model 5 HR(95%CI)	Model 6 HR(95%CI)
**Overall mortality**								
STP ratio	480	419(87.3)	1.12 (1.04-1.21)	1.10 (1.01-1.19)	1.13 (1.04-1.23)	1.12 (1.03-1.22)	1.17 (1.05-1.32)	1.17 (1.05-1.31)
Median-based urinary STP ratio category								
<1.29	217	180(43.0)	Ref	Ref	Ref	Ref	Ref	Ref
≥1.29	263	239(57.0)	1.41 (1.16-1.71)	1.33 (1.09-1.61)	1.35 (1.11-1.65)	1.29 (1.05-1.58)	1.17 (0.92-1.48)	1.18 (0.93-1.50)
**Cancer-specific mortality**								
STP ratio	480	391(81.5)	1.13 (1.04-1.22)	1.11 (1.02-1.21)	1.14 (1.05-1.24)	1.13 (1.04-1.24)	1.18 (1.05-1.32)	1.17 (1.05-1.32)
Median-based urinary STP ratio category								
<1.29	217	165 (42.2)	Ref	Ref	Ref	Ref	Ref	Ref
≥1.29	263	226 (57.8)	1.42 (1.16-1.74)	1.35 (1.10-1.65)	1.37 (1.12-1.69)	1.30 (1.05-1.60)	1.16 (0.91-1.47)	1.17 (0.91-1.49)

B. In the non-pulmonary NEC subgroup.

Variable	Total No.	Events (n%)	Model 1 HR(95%CI)	Model 2 HR(95%CI)	Model 3 HR(95%CI)	Model 4 HR(95%CI)	Model 5 HR(95%CI)	Model 6 HR(95%CI)
**Overall mortality**								
STP ratio	279	167(59.9)	1.08 (0.93-1.27)	1.15 (0.98-1.36)	1.20 (1.02-1.43)	1.12 (0.93-1.34)	1.15 (0.93-1.43)	1.20 (0.96-1.50)
Median-based urinary STP ratio category								
<1.29	160	92(55.1)	Ref	Ref	Ref	Ref	Ref	Ref
≥1.29	119	75(44.9)	1.09 (0.81-1.48)	1.19 (0.87-1.62)	1.24 (0.90-1.70)	1.14 (0.82-1.58)	1.23 (0.84-1.80)	1.26 (0.86-1.85)
**Cancer-specific mortality**								
STP ratio	279	158(56.6)	1.11 (0.95-1.30)	1.18 (1.00-1.38)	1.24 (1.04-1.46)	1.15 (0.96-1.38)	1.19 (0.96-1.48)	1.22 (0.98-1.51)
Median-based urinary STP ratio category								
<1.29	160	85(53.8)	Ref	Ref	Ref	Ref	Ref	Ref
≥1.29	119	73 (46.2)	1.14 (0.84-1.56)	1.23 (0.90-1.69)	1.30 (0.94-1.80)	1.21 (0.87-1.69)	1.30 (0.88-1.93)	1.31 (0.89-1.94)

This table presents the hazard ratios (HR) and 95% confidence intervals (CI) for the association between the urinary sodium-to-potassium (STP) ratio and both overall mortality and cancer-specific mortality among 759 patients with neuroendocrine cancer (NEC), stratified by primary tumor site. The study population was divided into pulmonary NEC and non-pulmonary NEC subgroups. Among the 759 participants, 480 patients had pulmonary NEC and 279 patients had non-pulmonary NEC. In the pulmonary NEC subgroup, 217 participants had an STP ratio <1.29 and 263 participants had an STP ratio ≥1.29. During follow-up, 419 overall mortality events and 391 cancer-specific mortality events were observed among patients with pulmonary NEC. In the non-pulmonary NEC subgroup, 167 overall mortality events and 158 cancer-specific mortality events were observed.

The data were adjusted across six different models. The HRs for overall mortality and cancer-specific mortality are provided for both the continuous STP ratio and the median-based binary classification of STP ratio, with STP <1.29 as the reference group. In the fully adjusted Model 6, the association between continuous STP and mortality outcomes was more evident among patients with pulmonary NEC. In non-pulmonary NEC, the HRs showed a similar positive direction but did not reach statistical significance, likely due to the smaller sample size and wider confidence intervals. Formal interaction analyses did not show significant effect modification by primary tumor site, with P for interaction = 0.567 for overall mortality and P for interaction = 0.648 for cancer-specific mortality. These findings suggest that the association between urinary STP ratio and mortality outcomes remained observable in pulmonary NEC, while no statistically significant difference was detected between pulmonary and non-pulmonary NEC.

Model 1 was the crude model; Model 2 was adjusted for age at diagnosis and gender; Model 3 was adjusted for Model 2 plus BMI, diabetes, smoking history, and cardiovascular and cerebrovascular diseases, including angina, stroke, and hypertension; Model 4 was adjusted for Model 3 plus histology, surgical history, and merging/combined other malignant tumors; Model 5 was adjusted for Model 4 plus glucose, HbA1c, HDL, IGF-1, triglyceride, cholesterol, urate, creatinine, C-reactive protein, urea, and albumin; Model 6 was adjusted for Model 5 plus utilization of diuretics and sodium intake. Sodium intake refers to additional salt added to food and does not include salt used in cooking or salt contained in the food itself.
