# Supplementary material for: Association of urinary sodium and potassium excretion with outcomes of neuroendocrine cancer
Source: Front Oncol. 2026 Aug 12;16:1866503. doi: 10.3389/fonc.2026.1866503 (PMC13508161; doi:10.3389/fonc.2026.1866503)
Supplement: Supplementary file 2 [file Image1.pdf]

Supplementary Figure 1.

Percentile distribution of Urinary Sodium and Potassium Excretion.

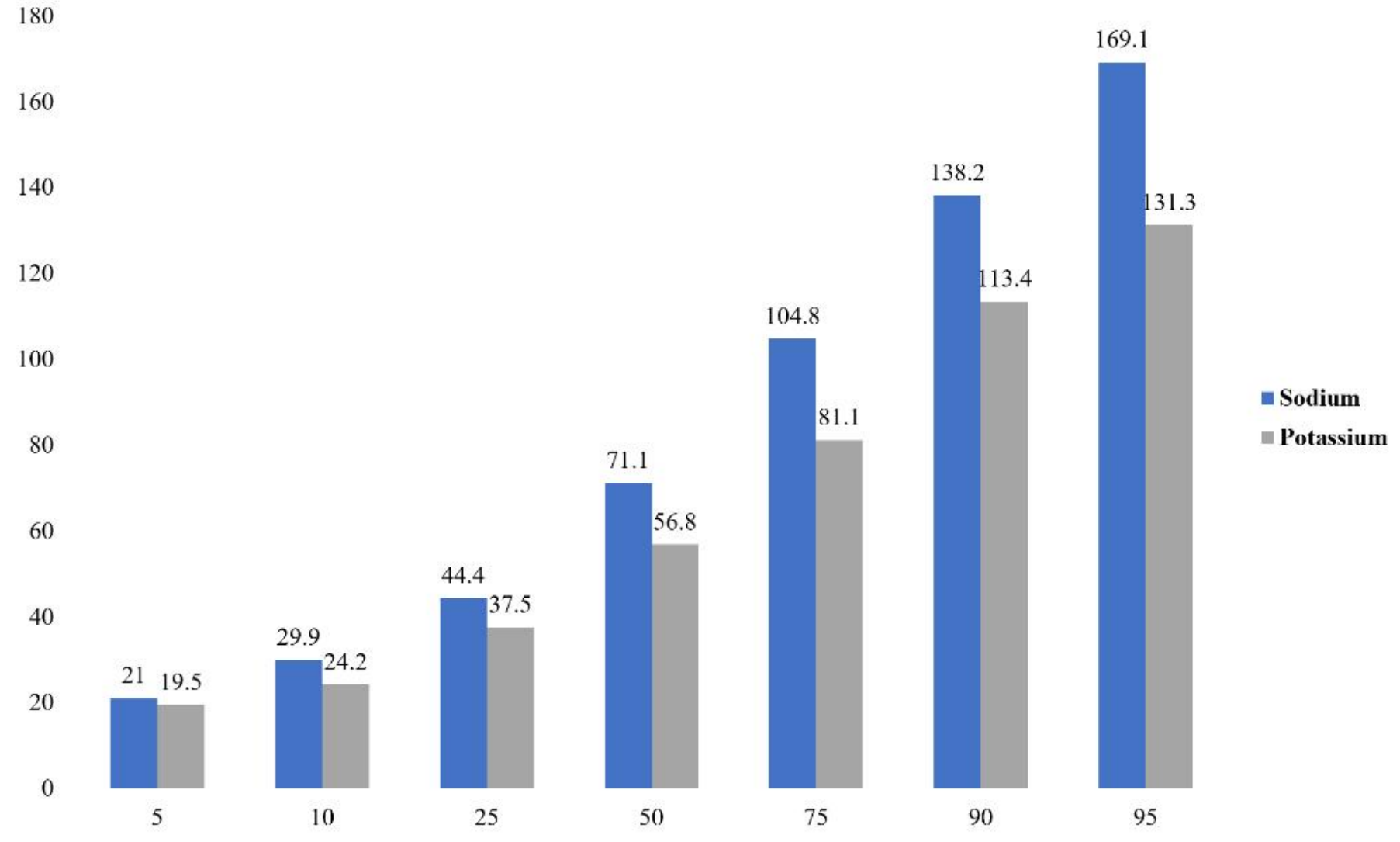

This bar chart displays the percentile distribution of urinary sodium (blue) and potassium (gray) excretion among the participants. The percentiles shown include the 5th, 10th, 25th, 50th, 75th, 90th, and 95th percentiles. For each percentile, the excretion values of sodium and potassium are presented to compare their distribution across the population.
